# Team-based care improves quality of diabetes care -Family Practice Integrated Care Project in Taiwan

**DOI:** 10.1186/s12875-020-01284-w

**Published:** 2020-10-15

**Authors:** Jerry Che-Jui Chang, Shinn-Jang Hwang, Tzeng-Ji Chen, Tai-Yuan Chiu, Hsiao-Yu Yang, Yu-Chun Chen, Cheng-Kuo Huang, Chyi-Feng Jan

**Affiliations:** 1 Department of Family Medicine, National Taiwan University Hospital, No. 17 Xuzhou Road, Taipei, 100 Taiwan; 2 Taiwan Medical Association, Taipei, Taiwan; 3 Taiwan Association of Family Medicine, Taipei, Taiwan; 4 Department of Family Medicine, Taipei Veterans General Hospital, Taipei, Taiwan; 5 School of Medicine, National Yang Ming University, Taipei, Taiwan; 6 Institute of Environmental and Occupational Health Sciences, National Taiwan University College of Public Health, Taipei, Taiwan; 7 Department of Public Health, National Taiwan University College of Public Health, Taipei, Taiwan; 8 Department of Environmental and Occupational Medicine, National Taiwan University Hospital and National Taiwan University College of Medicine, Taipei, Taiwan; 9 Dr. Cheng-Kuo Huang Clinic, Keelung, Taiwan

**Keywords:** Quality of health care, Family practice integrated care project, Delivery of health care, Policy, Primary health care, Diabetes mellitus

## Abstract

**Background:**

The Family Practice Integrated Care Project (FPICP) is a team-based program in Taiwan initiated in 2003. This study investigates the influence of FPICP on the quality of diabetes care.

**Methods:**

This population-based cohort study used Taiwan’s National Health Insurance Administration data on FPICP (fiscal year 2015–2016, with follow-up duration of one year). Participants included diabetic patients aged ≥30 in primary care clinics.

We used conditional logistic regression modeling of patient characteristics and annual diabetes examinations and compared FPICP participants with non-participating candidates. Main outcome measures included completion of annual diabetes examinations, including glycated hemoglobin (A1c), low-density lipoprotein (LDL), urine microalbumin (MAU), routine urinalysis (UR), and fundus examination (FE).

**Results:**

The sample included 298,208 FPICP participants and 478,778 non-participating candidates. After 1:1 propensity score matching, the examination completion rates for FPICP participants and non-participants, respectively, were 94.4% versus 93.6% in A1c, 84.2% versus 83.8% in LDL, 61.9% versus 60.1% in MAU, 59.2% versus 58.0% in UR, and 30.1% versus 32.4% in FE.

**Conclusion:**

Our findings indicate that a program like FPICP helps improve the quality of diabetes care through regular examinations of Alc, LDL, MAU, and UR.

## Background

Pay-for-performance (P4P) models, adopted by many countries worldwide, aim to improve healthcare quality by offering healthcare providers financial incentives based on performance indicators regarding the process of care, laboratory results, and clinical outcomes. P4P programs have been found to benefit the process of care [1]; however, they differ from one program to another in terms of design, and the results generated by certain P4P programs do not necessarily remain valid in others. Therefore, it is essential that researchers evaluate the performance of P4P programs on a case-by-case basis.

The Family Practice Integrated Care Project (FPICP) is a team-based integrated care program initiated in Taiwan in 2003, the year of severe acute respiratory syndrome (SARS) outbreak [2]. The SARS outbreak reflected the insufficiency of endemic disease preventive function in Taiwan’s communities. FPICP was launched to empower primary care physicians by providing additional resources and facilitating vertical integration between primary care clinics and community hospitals to build up a primary community care network [3].

The target population of FPICP are patients with multiple chronic diseases, patients with frequent use of outpatient department/clinic (OPD) care defined as ≥50 visits per year, and the elderly (age 65 years and over). Taiwan’s National Health Insurance Administration (NHIA) selects patients that incur higher medical costs among the target patients on a yearly basis to compile a list of FPICP candidates. For each patient, the primary care physician providing the most frequent healthcare service is invited to join the program by entering a Community Healthcare Group (CHCG) and registering the patient as an FPICP participant. Each CHCG is formed by five to ten clinics in a community with at least one community hospital or medical center in the same living area as their backup and mutual referral. Through continuous medical educational meetings and case discussions with hospital physicians every month, CHCGs intensify the communication and education among the colleagues. For patient care, the CHCGs and its referral hospital(s) work together to establish primary community care networks and receive FPICP incentives from the healthcare authorities. For physicians per participant per year, FPICP provides a payment of 250 points (1 point = NTD 0.9, the floating point value under the global budget scheme since 2001) as case management fee and 550 points bonus if performance passes certain quality assessments. The quality assessment indicators of FPICP are primarily preventive healthcare services, such as an influenza vaccination, adult health examination, and cancer screenings (Pap smear for cervical cancer, faecal immunochemical test for colon cancer, and oral cancer screening by inspection). A detailed list of quality assessment indicators is provided in Appendix 1.

Compared to those who did not join a CHCG, FPICP physicians provide team-based care to deliver patient-centered integrated health management, especially focusing on preventive care reminding, providing continuous care with telephone hotline consultation available for 24 h a day, and vertical integration between the primary care clinics and community hospitals. The vertical integration of FPICP includes mutual referral (clinic-to-hospital or hospital-to-clinic), shared healthcare information, regular medical educational meetings and case discussion meetings between primary care clinics and community hospitals. Once an FPICP participating patient was hospitalized, the family physician of primary care clinic also has a formal channel to participate in the patient round.

One of the paramount roles of primary care professionals is to handle the ever-increasing number of people with diabetes. According to the statistics released by Taiwan’s Health Promotion Administration (HPA) in 2016, the prevalence of diabetes in people aged 18 years or older was 11.8%, and there are around 2,275,267 diabetes patients in Taiwan, with an estimated increase of 25,000 new cases every year [4].

In this study, we investigated whether FPICP as a team-based integrated care program was able to improve the process of care in managing diabetes. Since the completion rates of related examinations for diabetes care are the immediate outcomes expected, we hypothesized that FPICP participants reported a higher rate of completing their annual examinations of glycated hemoglobin (A1c), low-density lipoprotein (LDL), urine microalbumin (MAU), routine urinalysis (UR) and fundus examination (FE), as compared to the non-participants.

## Methods

We conducted a population-based cohort study, comparing the completion rates of A1c, LDL, MAU, UR and FE among FPICP participating and non-participating diabetic patients in Taiwan. The study was in compliance with the Strengthening the Reporting of Observational Studies in Epidemiology (STROBE) checklist of essential items (version 4) for cohort study [5].

### Setting

The study used data from the reimbursement database of Taiwan’s NHIA containing all NHIA-selected patients for FPICP enrolment in the 2015 fiscal year, followed for one year (April 2015–March 2016). The consulted database, which enrolled 99.6% of Taiwan’s population [6], covered comprehensive drug prescription files and original claim data for reimbursement.

### Target population

Among patients in primary care clinics eligible for FPICP in the 2015 fiscal year, those aged over 30 diagnosed with diabetes formed the target population of the study. The patients with diabetes were defined as those visited an outpatient department at least twice in one year using ICD-9-CM code 250.

These NHIA-selected candidates became FPICP participants if their primary care physicians enrolled them in the project. In our study, the FPICP participants and non-participants were matched 1:1 by propensity score using age, gender, comorbidities, and participation in the diabetes pay-for-performance program (DMP4P). A brief explanation of propensity-score matching in this study is shown in Appendix 2. According to our previous study, FPICP patients had slightly lower prevalence of several chronic diseases, including diabetes, hyperlipidaemia, heart failure, hypertension, coronary artery disease, peripheral vascular disease, systemic embolism, cerebral vascular disease, and chronic kidney disease [3]. The presence of these chronic conditions are related to diabetes or its complications (macrovascular or microvascular) [7], and would influence the physicians’ behavior of ordering related examinations. In addition, participation in the diabetes pay-for-performance program (DMP4P) also influences the completion rate of diabetes examination. Therefore, we included above-mentioned variables along with age and gender in the propensity score matching. The ICD-9 codes used in this study are provided in Table S1.

### Variables

Taiwan’s NHIA has recommended several examinations to primary care physicians as part of integrated diabetes care, including A1c, LDL, MAU, UR, FE, and diabetes foot examinations [8], all of which, excluding the diabetes foot examination, are included in the NHIA reimbursement database. The care plan for diabetic patients should include the following key points about examination frequency [7, 8]:

- at least once every six months for A1c;
- once every year for blood lipid profiles, including total cholesterol, high-density lipoprotein, LDL and triglyceride;
- once every two years for eyes fundus examination;
- once a year for urine microalbumin;
- and at least once every year for a foot examination.

In our study, the outcomes of interest were the completion rates of the examinations of A1c, LDL, MAU, UR and FE among diabetic patients. The end date of follow-up was the date of death or March 31, 2016, whichever was earlier. The intervention of interest was the enrolment in FPICP. The participation in DMP4P, a major P4P program for diabetic patients since 2001, was considered a confounding factor in this study because of this existing program offering to monitor and rewards for diabetes-related care. Other factors regarded as potential confounders included age, gender, monthly income, region of residence, and comorbidities. Monthly income and region of residence were based on the data of Registry for Beneficiaries obtained upon the enrolment of a subject in the NHI program. Comorbidities were assessed using the Charlson Comorbidity Index (CCI) [9]. We defined the diagnosis of a comorbidity as receiving no less than twice of the same diagnosis within one year based on the ICD-9-CM codes as indicated by the physician claims data. The technical part of CCI calculation was based on the open-source SAS scripts published by the Health Care Delivery Research at the National Cancer Institute of the United States [10]. We have also made the SAS scripts of this study in the supplement (Appendix 3 and 4).

We converted the quantitative variables into categorical ones. “Elderly” was defined as patients aged 65 years or older, which is consistent with the WHO definition [11]. Monthly income was categorized by tertiles. Residential region codes were transformed into three levels of urbanization according to Taiwan NHRI publications (Appendix 5), with level 1 referring to the “most urbanized” and level 3 the “least urbanized” communities [12]. Increased comorbidity score was defined as CCI of 5 or greater, as suggested in Charlson’s study [9].

### Statistical analysis

Values were presented as either percentages or arithmetic means with standard deviations in descriptive analyses, and a chi-square test was conducted to compare categorical variables. Residual imbalance on individual covariate could still present after propensity-score matching. Therefore, the clinically important covariates used in the matching process were included in the final regression model to further adjust for potential confounding effects. For the matched cohort, conditional logistic regressions (conditional on the number of the cases in each stratum of a matched cohort) were performed to calculate the odds ratios (OR) of completing annual A1c, LDL, MAU, UR, and FE examinations on the influences of FPICP participation. Age (in categories), gender, comorbidities, and participation in DMP4P were included as independent variables in the model. A brief explanation of conditional logistic regression in this study is shown in Appendix 2.

A subgroup analysis was performed after reviewing the main study results. The coexistence of a diabetes care program (i.e. DMP4P) could limit the influence of FPICP. Although the confounding effect of DMP4P was adjusted it in the propensity score matching, we further explored the influence of FPICP on patients with or without DMP4P. Therefore, we categorized the matched cohort into two subgroups based on the participation in DMP4P, and compared the completion rates of annual diabetes examinations by participation in FPICP for each subgroup. A subsequent estimation of the odds ratios of completing annual diabetes examinations on participation of both FPICP and DMP4P was performed using conditional logistic regressions with an interaction term of both programs (FPICP * DMP4P). Age, gender, and comorbidities were included to adjust the odds ratios.

A two-tailed *P* value of 0.05 was considered statistically significant. All statistical analyses were conducted using SAS software (version 9.4; SAS Institute, Cary, NC).

## Results

**Fig. 1:**
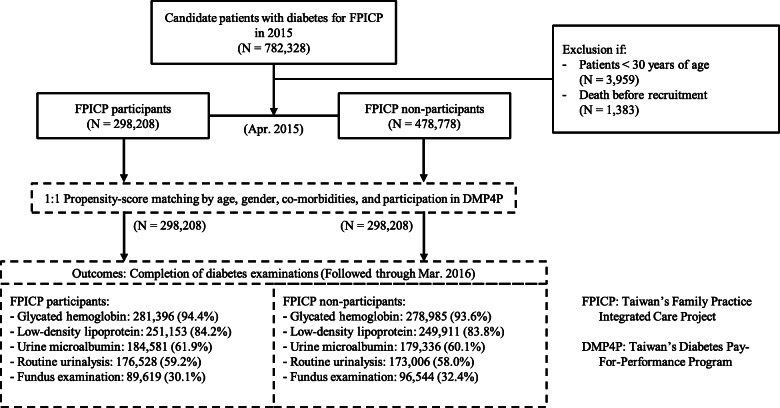
Data collection flowchart. The flowchart presents the numbers of patients at each steps of the study

Figure 1 illustrates the flowchart and results of data collection of the study. Of the 782,328 diabetic patients selected as FPICP candidates in 2015, there were 298,208 FPICP participants and 478,778 FPICP non-participants, excluding patients aged under 30 years and who passed away before enrolment. After 1:1 propensity score matching by age, gender, comorbidities, and participation in DMP4P, there were 298,208 FPICP participants and the same number of FPICP non-participants. Among them, 81,845 FPICP participants and 79,468 non-participants were also recruited into the DMP4P in Taiwan respectively.

### Descriptive data

Table 1 summarizes the characteristics of the study participants. Of all FPICP participants and non-participants, 53% were female, 66% were aged 50–75 years, and 5% had high comorbidities (CCI > 2). The differences were small between FPICP participants and non-participants in terms of monthly income (27% in the low-income category for both groups) and urbanization level of residence (33.2% versus 34% in the low-urbanization category).

**Table 1:** Characteristics of study subjects at enrolment

	FPICP Participants ***N*** = 298,208	Non-participants ***N*** = 298,208
**Gender**		
Female	157,940 (53.0%)	158,493 (53.1%)
Male	140,268 (47.0%)	139,715 (46.9%)
**Age (year)** ^**a**^		
30–50	28,953 (9.8%)	28,993 (9.7%)
50–65	106,678 (35.8%)	106,022 (35.6%)
65–75	90,508 (30.4%)	91,495 (30.7%)
75–85	57,829 (19.4%)	57,551 (19.3%)
over 85	14,240 (4.8%)	14,147 (4.7%)
**DMP4P**		
Participants	81,845 (27.5%)	79,468 (26.7%)
Non-participants	216,363 (72.6%)	218,740 (73.4%)
**Charlson Comorbidity Index**		
High (> 2)	15,619 (5.2%)	15,507 (5.2%)
Low (0–2)	282,589 (94.8%)	282,701 (94.8%)
**Monthly Income** ^**b c**^		
Level 1 (high)	98,821 (33.1%)	97,892 (32.8%)
Level 2 (medium)	118,804 (39.8%)	119,782 (40.2%)
Level 3 (low)	80,583 (27.0%)	80,534 (27.0%)
**Urbanization** ^**c**^		
Level 1 (high)	60,818 (20.7%)	68,806 (23.5%)
Level 2 (medium)	135,654 (46.1%)	124,362 (42.5%)
Level 3 (low)	97,509 (33.2%)	99,528 (34.0%)

*DMP4P* Taiwan’s Diabetes Pay-For-Performance Program; *FPICP* Taiwan’s Family Practice Integrated Care Project

^a^Age at enrollment (April 2015)

^b^Counted in New Taiwan dollar (NTD)

^c^Categorized by tertiles

### Main results

**Fig. 2:**
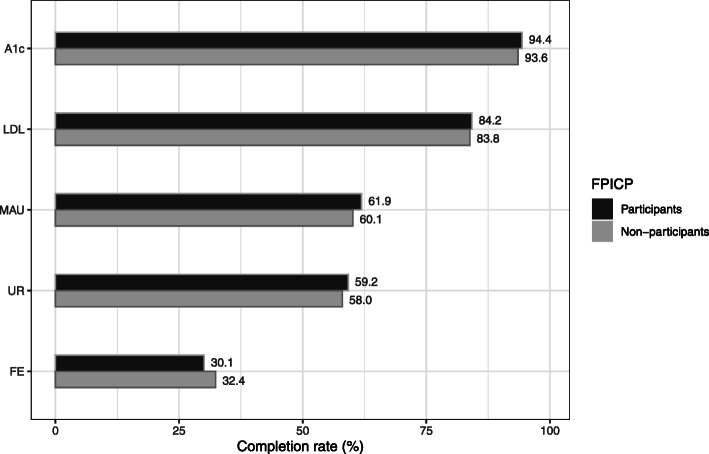
Completion rates of annual diabetes examinations, by participation of FPICP. The table and horizontal bar plot present the completion rates of annual diabetes examinations by participation of FPICP. Number of FPICP Participants: 298,208. Number of non-participants: 298,208. FPICP, Taiwan’s Family Practice Integrated Care Project

Figure 2 shows the completion rates of annual diabetes examinations for our study cohort, tested by the chi-squared test. For FPICP participants and non-participating candidates, the completion rates were respectively 94.4% versus 93.6% in A1c (*p* < 0.001), 84.2% versus 83.8% in LDL (*p* < 0.001), 61.9% versus 60.16% in MAU (*p* < 0.001), 59.2% versus 58.0% in UR (*p* < 0.001), and 30.1% versus 32.4% in FE (*p* < 0.001).

**Fig. 3:**
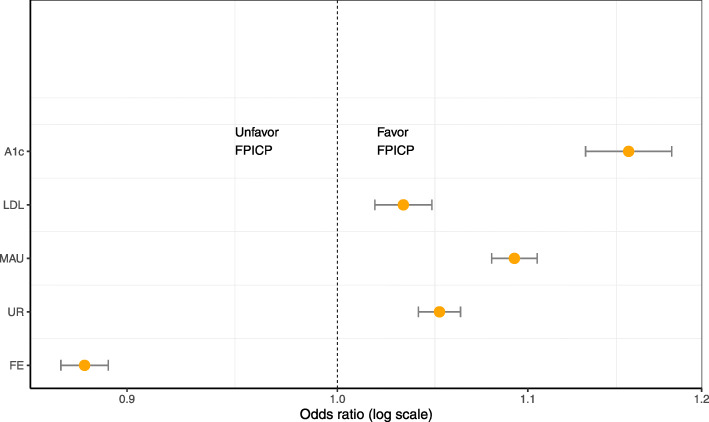
Associations between FPICP and diabetes process of care. The table and horizontal error bars present the adjusted odds ratios of completing annual diabetes examinations on participation of FPICP. The odds ratios and 95% confidence interval (in parentheses and the error bars) were estimated using conditional logistic regression. Other independent variables for adjusted odds ratios include age, gender, and comorbidities. FPICP, Taiwan’s Family Practice Integrated Care Project

The associations between FPICP and completion rates of diabetes examinations were studied using conditional logistic regression, with adjustments for age, gender, comorbidities, and participation in DMP4P (Fig. 3). The adjusted odds ratios of diabetes examinations in FPICP participants were 1.16 (1.13–1.18) in A1c, 1.03 (1.02–1.05) in LDL, 1.09 (1.08–1.11) in MAU, 1.05 (1.04–1.06) in UR, and 0.88 (0.87–0.89) in FE.

### Subgroup analysis

**Fig. 4:**
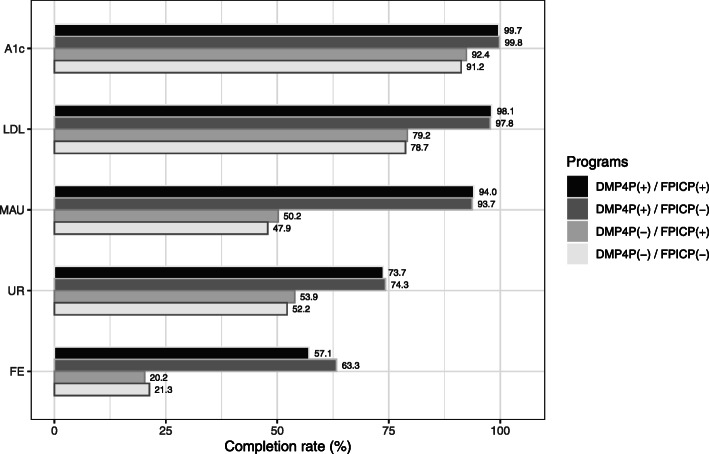
Completion rates of annual diabetes examinations, by participation of DMP4P and FPICP. The table and horizontal bar plot present the completion rates of annual diabetes examinations by participation of both DMP4P and FPICP. DMP4P, Diabetes Pay-for-performance program; FPICP, Taiwan’s Family Practice Integrated Care Project

Figure 4 shows the completion rates of annual diabetes examinations, categorized by participation in DMP4P and FPICP. Among patients without participation in any programs, the baseline completion rates of annual diabetes examinations were respectively 91.2% (A1c), 78.7% (LDL), 47.9% (MAU), 52.2% (UR), and 21.3% (FE). Among patients participating in only FPICP, the completion rates were 92.4% (A1c), 79.2% (LDL), 50.2% (MAU), 53.9% (UR), and 20.2% (FE), with slight increase except for the FE. For patients participating in DMP4P, the completion rates of annual diabetes examinations showed a significant increase (ranging from 7.3 to 45.9%) regardless of participation in FPICP.

The influences of these two programs on completion of diabetes examinations were also examined (Table 2). For DMP4P participation, the adjusted odds ratio of diabetes examinations was the most prominent, yielding 51.48 (43.88–60.39) in A1c, 11.95 (11.39–12.53) in LDL, 16.33 (15.85–16.82) in MAU, 2.62 (2.57–2.67) in UR, and 6.35 (6.24–6.46) in FE respectively. For FPICP participation, the adjusted odds ratio of diabetes examinations was 1.17 (1.15–1.20) in A1c, 1.03 (1.01–1.04) in LDL, 1.10 (1.09–1.11) in MAU, 1.07 (1.06–1.08) in UR, and 0.94 (0.92–0.95) in FE respectively. Furthermore, the interactions between FPICP and DMP4P on diabetes examinations were examined, and the the odds ratios were 0.52 (0.42–0.63) in A1c, 1.11 (1.04–1.19) in LDL, 0.96 (0.92–1.00) in MAU, 0.91 (0.89–0.93) in UR, and 0.83 (0.81–0.85) in FE respectively.

**Table 2:** Associations between pay-for-performance programs and diabetes process of care

	Adjusted Odds ratio	
	FPICP	DMP4P
**Glycated haemoglobin (A1c)**	1.17 (1.15–1.20)	51.48 (43.88–60.39)
**Low-density lipoprotein (LDL)**	1.03 (1.01–1.04)	11.95 (11.39–12.53)
**Urine microalbumin (MAU)**	1.10 (1.09–1.11)	16.33 (15.85–16.82)
**Routine urinalysis (UR)**	1.07 (1.06–1.08)	2.62 (2.57–2.67)
**Fundus examination (FE)**	0.94 (0.92–0.95)	6.35 (6.24–6.46)

The odds ratios and 95% confidence interval (in parentheses) were estimated with an interaction term (FPICP * DMP4P) using multiple logistic regressions. Other independent variables for adjusted odds ratios include age, gender, and comorbidities

*DMP4P* Diabetes Pay-for-performance program, *FPICP* Taiwan’s Family Practice Integrated Care Project

## Discussion

### Summary

In this study, our hypothesis was that FPICP as a modified P4P program helped to improve the completion rate of annual examinations for diabetic patients. Our study results show that FPICP participants in Taiwan reported a higher rate of completing A1c, LDL, MAU, and UR examinations. However, a significant decrease in the completion rate of FE in FPICP participants is noted. In brief, our hypothesis is supported by the results (Fig. 3), except for the screening of diabetic retinopathy.

### Strengths and limitations

The strengths of our study lie in its generalisability based on nationwide cohort and the high homogeneity between the FPICP participants and non-participants. The NHIA database provided a sufficient number of observations and the results were marked with a substantial degree of external validity. The target population was composed exclusively of FPICP candidates meeting the NHIA selection criteria (i.e., multiple chronic diseases, frequent users of OPD care, or the elderly). We also performed propensity score matching to further increase the homogeneity. To the best of our knowledge, this is the first population-based study to investigate the influences of FPICP on the completion of annual diabetes examinations.

Our study, however, is marked with several limitations. First, our study experienced a difficulty common in using the data obtained from databases for reimbursement purpose: information about lifestyle and personal behavior, such as smoking, body mass index, and dietary factors, could not be extracted from the insurance database. These factors affect the disease status of diabetes, and could be related to the adherence and communication between patient and doctor. Missing the information could bias the study results by overestimating the influence of FPICP if the participants had a higher level of health literacy and adherence, and by underestimating if the situation was the opposite. Second, since we used ICD-9-CM codes to identify diagnoses and comorbidities, our estimates may overestimate the true prevalence, as physicians might make tentative diagnoses based on their impressions. Third, we were unable to compare the quality of care between participants and non-participants before the FPICP in 2015; therefore, higher rates of proper checkups for diabetic patients might also result from the fundamental differences in healthcare providers of these two groups of patients.

### Interpretation of the findings

In Taiwan, especially in the primary care clinical setting, physicians have to take declaration for payment into consideration in preparation for data and record inspection by NHIA. The inspection frequency depends on whether the data and records check out. Compared to total cholesterol or triglyceride, LDL may be less frequently checked in the primary care setting because of the availability and deletion by NHIA. MAU is also less available in community care compared to urine protein analysis. Instead, urine protein or routine urinalysis is used to replace MAU. That is a major reason why A1c had the best completion rate in our study. FPICP grants an incentive made up of 10% weighting of quality assessment on preventable hospitalization for complications related to chronic diseases, including diabetes (Appendix 1). Although not direct incentives, it still makes sense for FPICP primary care clinics to follow the guidelines and NHIA recommendations for diabetes care more closely.

The small differences in examination rates lead to a discussion on the clinical significance (e.g. difference of 0.8% for A1c). Although significant in statistics, the influences of FPICP on the completion rates of diabetes examinations are small, as the program was not solely focused on diabetes care. However, according to Taiwan’s National Health Research Institutes, the number of diabetic patients are 2.2 millions in 2014, accounting for 9.3% of Taiwanese population [13]. Compared to 840 thousand diabetic patients in 2000, the population has increased 2.6 times. In this sense, 1 % difference in completion rate affects 22 thousand patients, and the population is growing approximately 7% per year. Therefore, we suggest that the positive impacts of FPICP on the completion rates on diabetes examinations are meaningful to the public health experts and the policy decision makers.

The opposite finding was noted regarding the regular FE examination, which reported a lower completion rate among patients recruited into FPICP. This finding is likely to be related to the expertise and equipment needed to screen for diabetic retinopathy, which is usually performed by ophthalmologists. To further support this, we compared the medical specialty of the FPICP member physicians to that of local practicing physicians in total. FPICP member physicians were more likely to be specialized in family medicine (32.8%), internal medicine (19.0%), and pediatrics (14.1%). In contrast, ophthalmologists accounted for only 3.8% of FPICP physicians, compared to 6.3% among all local practicing physicians. A detailed comparison is shown in Table S3. In a study with a small sample size by Gill et al., eleven family physicians in Delaware assessed twenty-eight standardized patients with diabetes using nonmydriatic ophthalmoscope [14]. The mean sensitivity was 87% with a specificity of 57%, indicating that the technique of the family physicians was not sufficiently effective in screening for diabetic retinopathy. Likewise, primary care physicians in Taiwan who join FPICP might also be less familiar with ophthalmoscopy than they are with other diabetes examinations, thus resulting in a suboptimal completion rate in FE examination. This finding indicates that actions are needed to facilitate primary care referrals to local ophthalmologist’s clinics or using artificial intelligence-assisted devices that can help with the screening or diagnosis of diabetes retinopathy.

There might be fundamental differences between FPICP primary care clinics and their non-participating counterparts. For each year, the NHIA selects patients with more comorbidities and utilizing more medical resources and assigns them to their most frequently visited primary care clinics which need to register all assigned patients for FPICP, if they would like to join the yearly project. In other words, for NHIA-selected patients who are not enrolled in FPICP, their primary care clinics are those that do not apply for the yearly project. It is possible that what helps improve the quality of diabetes care are primary care clinics providing better diabetes care, and they are more inclined to join other P4P programs, rather than FPICP itself. The reasons for a primary care clinic to apply or not for FPICP merits further investigations.

In our subgroup analysis, it became clear that DMP4P yielded much higher odds ratios on completion of diabetes examinations in comparison with FPICP. The probable reasons for the differences were twofold. First, the DMP4P has direct quality assessment items on completion of diabetes examinations as DMP4P is disease-specific care, while FPICP is not. Second, physicians that participated in DMP4P can choose which patients are to be recruited, resulting in possible selection bias (a cherry-picking phenomenon) [1, 15], whereas the design of FPICP did not allow primary care clinics to select which patients to recruit.

The interactions between FPICP and DMP4P on the completion of diabetes examinations were observed. There was negative synergy between these two P4P programs on checkups of A1c, UR, and FE, which indicated that FPICP had less impact on DMP4P participants than on those non-participants. Possible explanation could be related to the competition on time resource of both physicians and patients who had multiple targets of quality of care to fulfill. Nevertheless, these findings on the interactions between two programs were not hypothesis-driven, and could be incidental without implications on health policies.

### Comparison with existing literature

FPICP accounts for approximately USD 40 million or two per-mille of Taiwan’s USD 20 billion health insurance budget every year; however, studies in peer-reviewed international journals exploring the effectiveness of this project remains limited, and none of them are quantitative [2, 16].

The completion rates of diabetes-related examinations vary among different studies in Taiwan [17–20]. The variety might be a result of the year of study, the level of hospitals/clinics providing services, the accessibility of medical resources, and the intervention of healthcare programs among the cohorts. In earlier studies conducted before 2005 by Tseng et al. and Hsu et al., the completion rate of A1c examination were 3.3% (data from clinics) and 51.6% respectively [17, 18]. These studies reported lower completion rates probably because of the lower availability of diabetes examination in that era. It is worth noting that the study by Tseng et al. shares the same setting of primary care clinics as our study, whereas most other studies we were able to identify report the completion rates of examinations at both clinics and hospitals together. One cross-sectional study on DMP4P participants in 2008 revealed 100 and 92% completion rate of A1c examination among enrolled and non-enrolled patients [19]. The perfect completion rate of A1c examination indicated the impressive effectiveness of DMP4P on enrolled patients. However, the arbitrariness of patient enrollment in DMP4P could also produce a selection bias in that study. Another study on participants of a diabetes management program in Changhua reported a 92.2% completion rate of A1c examination in 2012 [20]. According to that study, the result was better in the participants than in the general population, demonstrating the efficacy of the diabetes management program. A more detailed comparison is shown in Table S2.

### Implications for research

Our database included all diabetic patients nationwide eligible for the FPICP program in 2015, and followed through 2016. Since significant differences in the design of P4P programs and healthcare system among countries can arise, we assume that the results of this study are more applicable to healthcare systems based on the Beveridge Model, such as the ones in the United Kingdom and Scandinavia, or to those with a single-payer insurance scheme, such as the ones in Canada and South Korea. Taiwan’s experience of FPICP demonstrate that, for patients making frequent OPD visits, incentivizing primary care physicians with case management fee and bonus for quality performance helps to successfully promote preventive healthcare services and improve patients’ satisfaction [2], as well as improve holistic healthcare in terms of the process of care. The design of FPICP in Taiwan may serve as a reference for health care policy makers interested in launching an integrated primary care program with P4P features.

## Conclusion

FPICP as a team-based care program helps to improve the quality of diabetes care by raising the completion rates of examinations through team-based care and more intensive health management. However, for patients already participating in other goal-directed diabetes care programs (e.g. DMP4P), the quality improvement is limited. Future research may like to focus on examining the longer-term impacts of P4P programs and comparing the designs of the programs in terms of cost-effectiveness.

## Supplementary information

**Additional file 1: Table S1.** ICD codes used in this study. **Table S2.** Comparison of completion rates of diabetes examinations among diabetes cohorts in Taiwan. **Table S3.** Participation rate in FPICP among local practicing physicians by medical specialty. **Appendix 1.** Quality assessment indicators for CHCG. **Appendix 2.** A Brief Explanation of Propensity-Score Matching and Conditional Logistic Regression in this study. **Appendix 3.** SAS main script. **Appendix 4.** SAS modules scripts. **Appendix 5.** Level of urbanization according to the region code in Taiwan.

## Data Availability

The data that support the findings of this study are available from National Health Insurance Administration (Taiwan), but restrictions apply to the availability of these data, which were used under license for the current study, and so are not publicly available. Data are however available from the authors upon reasonable request and with permission of National Health Insurance Administration (Taiwan).

## Abbreviations

ADA: American Diabetes Association
A1c: glycated hemoglobin
CCI: Charlson Comorbidity Index
CHCG: Community HealthCare Group
CI: Confidence interval
DMP4P: Diabetes Pay-for-performance Program (Taiwan)
FE: fundus examination
FPICP: Family Practice Integrated Care Project (Taiwan)
HPA: Health Promotion Administration
ICD-9-CM: International Classification of Diseases, Ninth Revision, Clinical Modification
LDL: Low-density lipoprotein
MAU: Urine microalbumin
NHIA: National Health Insurance Administration (Taiwan)
NHRI: National Health Research Institutes (Taiwan)
NTD: New Taiwan dollar
OPD: Outpatient department / clinic
OR: Odds ratio
P4P: Pay-for-performance
STROBE: Strengthening the Reporting of Observational Studies in Epidemiology
UR: Routine urinalysis
USD: US dollar
WHO: World Health Organization
