# Supplementary material for: Team-based care improves quality of diabetes care -Family Practice Integrated Care Project in Taiwan
Source: BMC Fam Pract. 2020 Oct 15;21:209. doi: 10.1186/s12875-020-01284-w (PMC7561235; doi:10.1186/s12875-020-01284-w)
Supplement: Supplementary file 1 — Additional file 1: Table S1. ICD codes used in this study. Table S2. Comparison of completion rates of diabetes examinations among diabetes cohorts in Taiwan. Table S3. Participation rate in FPICP among local practicing physicians by medical specialty. Appendix 1. Quality assessment indicators for CHCG. Appendix 2. A Brief Explanation of Propensity-Score Matching and Conditional Logistic Regression in this study. Appendix 3. SAS main script. Appendix 4. SAS modules scripts. Appendix 5. Level of urbanization according to the region code in Taiwan. [file 12875_2020_1284_MOESM1_ESM.docx]

# Supplement

## Table S1. ICD codes used in this study

|  | Disease | ICD-9 |
| --- | --- | --- |
| Diagnosis for inclusion criteria | Diabetes | 250 |
| Comorbidities for propensity-score matching | Hyperlipidaemia | 403.01 , 403.11 , 403.91 , 404.02 , 404.03 , 404.12 , 404.13 , 404.92 , 404.93 , 585 , V45.1 , V56.0 , V56.8 |
|  | Heart failure | 428 |
|  | Hypertension | 401 , 402 , 403 , 404 |
|  | Coronary artery disease | 414 |
|  | Peripheral vascular disease | 441 , 443.9 , 785.4 , V43.4 |
|  | Diabetes complications | 250.4 , 250.5 , 250.6 |
|  | Systemic embolism | 444 |
|  | Cerebral vascular disease | 430 , 431 , 432 , 433 , 434 , 435 , 436 , 437 , 438 |
|  | Chronic kidney disease | 272 |
| Diagnoses for Charlson comorbidity index | Acute myocardial infarction | 410, 412 |
|  | Heart failure | 428 |
|  | Peripheral vascular disease | 441 , 443.9 , 785.4 , V43.4 |
|  | Cerebral vascular disease | 430 , 431 , 432 , 433 , 434 , 435 , 436 , 437 , 438 |
|  | Dementia | 290 |
|  | Pulmonary disease | 490, 491, 492, 493, 494, 495, 496, 500, 501, 502, 503, 504, 505 |
|  | Connective tissue disorder | 710.0, 710.1, 710.4, 714.0, 714.1, 714.2, 714.81, 517.1, 725 |
|  | Peptic ulcer | 531, 532, 533, 534 |
|  | Liver disease | 571.2, 571.4, 571.5, 571.6 |
|  | Diabetes | 250 |
|  | Diabetes complications | 250.4 , 250.5 , 250.6 |
|  | Paraplegia | 342, 344.1 |
|  | Renal disease | 582, 583.0, 583.1, 583.2, 583.3, 583.5, 583.6, 583.7, 583.4, 585, 586, 588 |
|  | Cancer | 14, 15, 16, 18, 170, 171, 172, 174, 175, 176, 179, 190, 191, 192, 193, 194, 195.0, 195.1, 195.2, 195.3, 195.4, 195.5, 195.8, 200, 201, 202, 203, 204, 205, 206, 207, 208 |
|  | Metastatic cancer | 196, 197, 198, 199.0, 199.1 |
|  | Severe liver disease | 572.2, 572.3, 572.4, 572.8 |
|  | HIV | 042, 043, 044 |
| **Diagnosis definition: Number of outpatient visit ≥2 in one year** | | |

## Table S2. Comparison of completion rates of diabetes examinations among diabetes cohorts in Taiwan

| **Study** | **Cohort definition** | **Level of hospital** | **Annual performance rate** |
| --- | --- | --- | --- |
| Tseng, 2006  ([1](#_ENREF_1)) | 2,224 diabetic patients were sampled out randomly from claims data of National Health Insurance program of Taipei Branch in 2001 | Clinics | A1c, 3.3%;  LDL, 7.6%;  UR, 4.3%;  FE, 3.5% |
| Hsu, 2012  ([2](#_ENREF_2)) | 1,462 patients newly-diagnosed with diabetes from a representative cohort aged > 20 years and free of diabetes (n = 600,662) in 2000 followed up until 2005 | Medical centres, regional hospitals, local hospitals and clinics | A1c, 51.6%;  LDL, 27.8%;  MAU, 6.8%;  UR, 11.7%;  FE, 15.3%  (assessed one year within the diagnosis of diabetes) |
| Lai, 2013  ([3](#_ENREF_3)) | 146,467 patients enrolled in the nationwide DMP4P program in 2008 (cross-sectional study) | Medical centres, regional hospitals, local hospitals and clinics | A1c, 100%;  LDL, 80%;  UR, 77%;  FE, 67% |
| Yeh, 2014  ([4](#_ENREF_4)) | 3,150 diabetes patients enrolled in a diabetes management program in Changhua, Taiwan, followed from 2008 to 2012 | Medical centres, regional hospitals, local hospitals and clinics | A1c, 92.2%;  LDL, 86.6%;  MAU, 72.2%;  FE, 58.5%  (in 2012) |

**Reference:**

1. Tseng F-Y, Lai M-S, Syu C-Y, Lin C-C. Professional accountability for diabetes care in Taiwan. Diabetes research and clinical practice. 2006;71(2):192-201.

2. Hsu C-C, Lee C-H, Wahlqvist ML, Huang H-L, Chang H-Y, Chen L, et al. Poverty increases type 2 diabetes incidence and inequality of care despite universal health coverage. Diabetes care. 2012:DC_112052.

3. Lai C-L, Hou Y-H. The association of clinical guideline adherence and pay-for-performance among patients with diabetes. Journal of the Chinese Medical Association. 2013;76(2):102-7.

4. Yeh Y-P, Chang C-J, Hsieh M-L, Wu H-T. Overcoming disparities in diabetes care: eight years' experience changing the diabetes care system in Changhua, Taiwan. Diabetes research and clinical practice. 2014;106:S314-S22.

## Table S3. Participation rate in FPICP among local practicing physicians by medical specialty

| **Specialty** | **FPICP members** | **Numbers of local practicing physicians** | **Participation rate** |
| --- | --- | --- | --- |
|  | N = 4,124 | N = 16,749 | **in FPICP** |
| Family medicine | 1,352 (32.8%) | 2,041 (12.2%) | 66.2% |
| Internal medicine | 785 (19.0%) | 2,291 (13.7%) | 34.3% |
| Pediatrics | 581 (14.1%) | 1,795 (10.7%) | 32.4% |
| Otolaryngology | 468 (11.3%) | 1,662 (9.9%) | 28.2% |
| OBGYN | 336 (8.1%) | 1,006 (6.0%) | 33.4% |
| Opthalmology | 156 (3.8%) | 1,048 (6.3%) | 14.9% |
| Orthopedics | 101 (2.4%) | 337 (2.0%) | 30.0% |
| Surgery | 88 (2.1%) | 670 (4.0%) | 13.1% |
| PM&R | 86 (2.1%) | 415 (2.5%) | 20.7% |
| Dermatology | 63 (1.5%) | 787 (4.7%) | 8.0% |
| Psychiatry | 51 (1.2%) | 355 (2.1%) | 14.4% |
| Urology | 16 (0.4%) | 49 (0.3%) | 32.7% |
| Others | 41 (1.0%) | 4,293 (25.6%)† | 1.0% |

† Including 3,768 physicians without medical specialty.

FPICP, Family Practice Integrated Care Project; OBGYN, obstetrics and gynecology;

PM&R, physical medicine and rehabilitation.

# Appendix 1. Quality assessment indicators for CHCG

CHCG: Community HealthCare Group

NHIA: National Health Insurance Administration (Taiwan)

For each scoring item that fails, the score is calculated by the proportion of achievement of the benchmark. The quality assessment is passed if the total score reaches 90 out of 100 (full score).

1. Management indicators (weight = 30%)
   1. Registration of all NHIA-assigned patients; must be 100%.
   2. Participation once a month in the case discussion seminar, the combined care clinic, the community healthcare education, or the ward round in backup hospital(s).
   3. Providing a twenty-four hour consultation hotline for NHIA-assigned patients (tested 6 times per year)
2. Clinical indicators (weight = 40%)
   1. Rate of emergency department visits (excluding trauma): should be lower than the median of all eligible patients selected by NHIA.
   2. Rate of hospitalization due to pneumonia, coronary artery disease, diabetes related complications, chronic obstructive pulmonary disease, or urinary tract infection: should be lower than the median of all eligible patients selected by NHIA.
   3. Rate of needle injection (excluding insulin injection and vaccination): should be lower than the median of all eligible patients selected by NHIA.
   4. Rate of antibiotics use: should be lower than the median of all eligible patients selected by NHIA.
3. Feedbacks from participants (weight = 30%)
   1. Patient satisfaction survey by phone: should be higher than 80/100
   2. Compliance with healthcare policies
      1. Rate of providing adult health examination for patients over 40: should be higher than the median of all eligible patients selected by NHIA.
      2. Rate of providing Pap smear for women over 30: should be higher than the median of all eligible patients selected by NHIA.
      3. Rate of providing influenza vaccination for patients over 65: should be higher than the median of all eligible patients selected by NHIA.

# Appendix 2. A Brief Explanation of Propensity-Score Matching and Conditional Logistic Regression in this study

Propensity score is the probability of a patient with certain characteristics being assigned to a treatment arm. Propensity score matching is a method of matching the treatment group and control group with an aim to reduce confounders as an alternative to a randomized control design. It converts the confounding factors into the propensity score using logistic regression to estimate the log-likelihood of receiving treatment on a given matrix of covariates, and then matches the treatment group to the control group based on the propensity score of each patient.

While logistic regression estimates the odds ratio of Y on X for unmatched cohort, conditional logistic regression is an extension of logistic regression, taking into account the strata produced by the matching process. It is conditional on the number of the cases in each stratum of a matched cohort; therefore, without assigning dummy variables to the strata, conditional logistic regression yields the odds ratio by maximizing the sum of the log-likelihoods of each stratum.

# Appendix 3. SAS main script

Filename: Main script.sas

Description: The SAS main script for the process of statistical analysis using SAS 9.4. The main script calls other SAS modules listed in Appendix 3.

/*

Time: April 2015 ~ March 2016

Patients: NHI beneficiaries with age >= 30, diabetes

Intervention: Patients registered in the Family Practice Integrated Care Project (FPICP)

Comparison: Candidates of Family Practice Integrated Care Project (FPICP) without registration

Outcome: checkup of glycated hemoglobin (A1c), low-density lipoprotein (LDL),

urine microalbumin (MAU), routine urinalysis (UR), and

fundus examination (FE)

Major confounding factor: Diabetes pay-for-performance program (DMP4P)

*/

/*OPTIONS settings*/

options nolabel nonumber nodate mprint;

/*Data library settings*/

libname data "\fm\data\DM"; /*directory for working data*/

libname pre "\fm\preprocessed"; /*directory for pre-processed dataset*/

libname m "\SAS"; /*directory for data input from NHI beneficiaries*/

libname source "\fm\source"; /*directory for data regarding drug input other than the NHI beneficiaries*/

/*Setting directory of SAS modules*/

%let basepath= \SAS modules\;

/* Constructing the "ID List" of target population

Input: d802 (List of candidates), d803 (List of participants), D201 (Registry for Beneficiaries) files;

Output: data.FPICP_enroll; marking the FPICP participants and original candidates*/

%include "&basepath.ID_list from D802.sas";

/* Output “ID List” data in budget year 2015

Prerequisite: data.FPICP_enroll

Grouping variable: participation (of FPICP)*/

**DATA** data.ID_list2015; SET data.FPICP_enroll;

if year(valid_date) = **2015** then output; **run**;

**PROC** **freq** data=data.ID_list2015;

title 'FPICP patients list 2015';

tables participation; **run**;

/*Selecting patients with diabetes*/

/* Prerequisite:

data.ID_list2015: FPICP candidates in 2015

pre.outpatient: Sorted claims data of outpatient department visits, with patient’s ID, budget year, and frequency of visits by diagnoses

ouput: data.id_list2015_dm_all (FPICP candidates with diabetes in 2015) */

**PROC** **SQL**; CREATE TABLE data.id_list2015_dm_all AS

select t1.*, t2.*

from data.ID_list2015 as t1 left join pre.outpatient(keep = ID_year DMP4P hypertension--charlson) as t2

on t1.id_year = t2.id_year

and t2.diabetes = **1**; /*Marking patients with diabetes*/

**QUIT**;

**DATA** data.id_list2015_dm_all; SET data.id_list2015_dm_all;

if DMP4P >= **1** then DMP4P_group = **1**; else DMP4P_group = **0**; /* the variable DMP4P denotes the frequency of OPD visits due to participation in DMP4P */

if diabetes = **1** then output; /*Selecting patients with diabetes*/

**PROC** **freq** data=data.id_list2015_dm_all;

title 'FPICP DM patients list 2015';

tables participation; **run**;

/* Data exclusion:

The exclusion criteria were:

- patients younger than 30,

- patients dropped out NHI program at the start

Input parameters:

enroll_list: target file containing the patient list

Outputs: data.ID_list_e after fulfilling the inclusion/exclusion criteria

*/

%include "&basepath.exclusion_major 4.1.sas";

%***exclusion***(data.id_list2015_dm_all);

/*Compute the probability for the propensity-score matching (PSM)*/

/* Perform the Logistic Regression */

/* Calculate and save propensity score */

/* Propensity score name = PROB */

**PROC** **LOGISTIC** DATA = data.ID_list_e;

CLASS sex(ref = '2') / param = ref; /*The variable sex is 1 for male, 2 for female*/

MODEL participation (event = '1') = sex age ins_amt DMP4P_group hyperlipidemia CHF hypertension CAD PVD diabetes_comp embolism CVD CKD

/* ins_amt, monthly income;

DMP4P_group, participation of DMP4P;

CHF, heart failure;

CAD, coronary artery disease;

PVD, peripheral vascular disease;

diabetes_comp, complications of diabetes;

embolism, systemic embolism;

CVD, cerebral vascular disease;

CKD, chronic kidney disease*/

/ SELECTION = none RISKLIMITS

LACKFIT RSQUARE PARMLABEL;

OUTPUT OUT = data.id_list_e_PSM prob=prob ;**RUN**;

**DATA** PSM_treated; SET data.id_list_e_PSM; /* FPICP participants with propensity score*/

IF participation = **1** then do;

rename prob = pscoreT;

idT = _n_;

output;

end;

DATA PSM_untreated; SET data.id_list_e_PSM; /* FPICP non-participants with propensity score*/

If participation = **0** then do;

rename prob = pscoreC;

idC = _n_;

output;

end; **run**;

/* Include the propensity score matching macro code */

%include "&basepath.PSMatching.sas";

/* Call Macro and Perform 1:1 Match */

%***PSMatching***(datatreatment=PSM_treated, /*treatment dataset*/

datacontrol=PSM_untreated, /*control dataset*/

method=NN, /*method parameter can be NN (nearest available neighbor), caliper or radius.*/

numberofcontrols=**1**, /*number of control matched per treatment*/

caliper=**0.01**, /*Caliper can be any number indicating the size of the caliper*/

replacement=no, /*(yes/no) A control unit could be selected more than once.*/

dataoutput = data.id_list_e); /*output dataset*/

/*Checking the numbers in FPICP participants and non-participants, status post PSM*/

**proc** **freq** data = data.id_list_e;

title 'FPICP DM patients post-PSM';

tables participation / norow nopercent; **run**;

/*Table 1.demographics: FPICP participants vs. non-participants

age (at the start date of the study)

sex (gender of the beneficiary)

income (monthly income in TWD)

fu_time: follow-up time

urban: level of urbanization (high, medium, low)

*/

/* Input parameters:

ID_list: target file containing the ID list

depend: dependent variable for grouping

Outputs: "Data.ID_list_e_t1" after producing the table 1 (demographics)

*/

%include "&basepath.demographic_analysis 4.1.sas";

%***demographic_analysis***(data.id_list_e, participation);

/*Check the relationship between Chalson score (CCI) and FPICP*/

**Proc** **Freq** data = data.ID_list_e_t1; tables Charlson * participation; **run**;

**Data** data.id_list_e_t1_ch; Set data.ID_list_e_t1; /*Define Charlson >= 5 as high CCI; cut-off ref: https://www.ncbi.nlm.nih.gov/pubmed/3558716*/

if not (Charlson > **0**) then Charlson = **0**; /* missing value --> 0*/

if Charlson < **5** then CCI = "low "; else CCI = "high";

**run**;

/*Check the relationship between levels of Chalson score (high CCI and low CCI) and FPICP*/

**proc** **freq**; tables CCI * participation / norow nopercent chisq; **run**;

/*Procedures before doing the regression*/

/*The unit of time-to-event is "year" in the regression*/

/*defining "elder" as age >= 65*/

/* Input parameters:

ID_list: target file containing the ID list

output_file: the name of the resulting output file for subsequent regression

Output: "data.before_regression"

*/

%include "&basepath.before_regression_simple 4.1.sas";

%***before_regression_simple***(data.id_list_e_t1_ch, data.before_regression);

/*Assessing quality of integrated DM care */

%include "&basepath.assess_oo_target1 DM 4.1.sas";

%***assess_oo_target1***();

%include "&basepath.assess_oo_target2 DM 4.1.sas";

%***assess_oo_target2***(data.before_regression, data.before_regression_oo, A1C);

%***assess_oo_target2***(data.before_regression_oo, data.before_regression_oo, Fundus);

%***assess_oo_target2***(data.before_regression_oo, data.before_regression_oo, MAU);

%***assess_oo_target2***(data.before_regression_oo, data.before_regression_oo, LDL);

%***assess_oo_target2***(data.before_regression_oo, data.before_regression_oo, Urine);

/*Completion = once per year*/

/*Comparing outcomes by logistic regression*/

/*Grouping into binomial variables (checkup once per year, or not)*/

**DATA** data.before_regression_log; SET data.before_regression_oo;

if (A1C >= round(time)) then A1C_group = **1**; else A1C_group = **0**;

if (Fundus >= round(time)) then Fundus_group = **1**; else Fundus_group = **0**;

if (MAU >= round(time)) then MAU_group = **1**; else MAU_group = **0**;

if (LDL >= round(time)) then LDL_group = **1**; else LDL_group = **0**;

if (Urine >= round(time)) then Urine_group = **1**; else Urine_group = **0**;

**RUN**;

/*Reporting numbers of outcome events or summary measures over time.*/

/*Parameters

ID_list: target file containing the ID list to calculate the of outcome events

event: the name of variable of interest (the event)

fu_time_year: the variable name containing the follow-up time (year)

*/

%include "&basepath.num_of_event_table 4.1.sas";

%***num_of_event_table***(data.before_regression_log);

%include "&basepath.Logistic_data 4.1.sas";

%***Logistic_data***(data.before_regression_log, A1C_group);

%***Logistic_data***(data.before_regression_log, Fundus_group);

%***Logistic_data***(data.before_regression_log, MAU_group);

%***Logistic_data***(data.before_regression_log, LDL_group);

%***Logistic_data***(data.before_regression_log, Urine_group);

# Appendix 4. SAS modules scripts

Filenames:

ID_list from D802.sas

Exclusion_major 4.1.sas

PSMatching.sas

Demographic_analysis 4.1.sas

Before_regression_simple 4.1.sas

Assess_oo_target1 DM 4.1.sas

Assess_oo_target2 DM 4.1.sas

Num_of_event_table 4.1.sas

Logistic_data 4.1.sas

Description: The SAS modules called by the main script as procedures in the analysis.

## ID_list from D802.sas

/* Constructing the patient list from FPICP enrollment*/

/*Keep only patients recruited in budget year 2010-2015*/

/*candidates of FPICP*/

**DATA** FPICP_candidates; /*from D802*/

SET m.d802;

/* variables:

ID: Beneficiary identification

HOSP_ID: assigned clinic

BRANCH_ID: Regional division

VALID_DATE: Date of enrollment as candidates

BASE_TYPE: indication for FPICP (code "6" means joined other P4P)

*/

Budget_year = year(VALID_DATE);

id_year = id || Budget_year;

if (**2010** <= budget_year <= **2015**) then output; /*Keep only patients recruited in budget year 2010-2015*/

KEEP Budget_year id_year ID BRANCH_ID HOSP_ID VALID_DATE BIRTHDAY SEX BASE_TYPE;

**run**;

**PROC** **sort**; by Budget_year id; **run**;

**DATA** FPICP_candidates; SET FPICP_candidates; by Budget_year id; /*each id could be enrolled once per budget year*/

if last.id then output;

**run**;

/*participants of FPICP*/

**DATA** FPICP_participants; /*from D803*/

SET m.d803;

/* variables:

PRSN_ID: assigned physician

CASE_DATE: Date of enrollment as participants

*/

Budget_year = year(CASE_DATE);

id_year = id || Budget_year;

KEEP Budget_year id_year ID BRANCH_ID BIRTHDAY PRSN_ID CASE_DATE SEX;

**run**;

**PROC** **sort**; by Budget_year id; **run**;

**DATA** FPICP_participants; SET FPICP_participants; by Budget_year id; /*each id could be enrolled once per budget year*/

if last.id then output;

**run**;

/*marking the participants of FPICP in the candidates ---> "data.FPICP_enroll"*/

**PROC** **SQL**; CREATE TABLE data.FPICP_enroll AS

SELECT t1.*, t2.PRSN_ID, case

when PRSN_ID is not missing then **1**

else **0**

end as participation

FROM FPICP_candidates as t1 left join FPICP_participants as t2

ON t1.id_year = t2.id_year;

**QUIT**;

/*demographic data from D201*/

**DATA** ID_List; set m.d201_2010

m.d201_2011

m.d201_2012

m.d201_2013

m.d201_2014

m.d201_2015

m.d201_2016; /*aggregate data from the Registry for beneficiaries (ID) */

Out_year = year(OUT_DATE)+**1**; /*year after drop-out date*/

id_out_year = id || Out_year;

keep ID BIRTHDAY SEX INS_ID STATUS INS_AMT HOME_ZIP_CODE ZIP_CODE OUT_DATE id_out_year;

/* variables:

INS_ID: Insurant identification

STATUS: status of insurance

INS_AMT: monthly income

HOME_ZIP_CODE: zip code of household registration

ZIP_CODE: zip code of residence address

OUT_DATE: date of cancellation of insurance

*/

**run**;

**proc** **sort** data = ID_List; by id; **run**; /*sort by id*/

**Data** ID_FirstID; set ID_List; by id; /*remove duplicates by extracting from the first entry by ID*/

if first.id then output; **run**;

**PROC** **SQL**; CREATE TABLE ID_LastID AS /*assess the date of death*/

SELECT ID, max(OUT_DATE) as OUT_DATE, STATUS

FROM ID_List

WHERE STATUS = '5' /*status 5 means death*/

GROUP BY ID; **QUIT**;

**PROC** **SQL**; CREATE TABLE data.ID_List(drop = x status) AS /*update the "ID_list" with the latest OUT_DATE*/

SELECT a.*, b.OUT_DATE

FROM ID_FirstID(rename=(OUT_DATE=x)) as a left join ID_LASTID as b

ON a.ID = b.ID; **QUIT**;

/*marking the demographics ---> "data.FPICP_enroll"*/

**PROC** **SQL**; CREATE TABLE data.FPICP_enroll AS

SELECT t1.*, t2.INS_ID, t2.STATUS, t2.INS_AMT, t2.HOME_ZIP_CODE, t2.ZIP_CODE, t2.OUT_DATE, t2.id_out_year

FROM data.FPICP_enroll as t1 left join data.ID_List as t2

ON t1.id = t2.id; **QUIT**;

**PROC** **freq** data=data.FPICP_enroll;

title 'FPICP patients list';

tables participation BASE_TYPE;

**run**;

## Exclusion_major 4.1.sas

/*start_date = valid_date: the date as FPICP candidates*/

/* Data exclusion:

The exclusion criteria were:

- patients younger than 30,

- patients dropped out NHI program at the start

Input parameters:

enroll_list: target file containing the patient list

Outputs: data.enroll_list_e after fulfilling the inclusion/exclusion criteria

*/

**%macro** exclusion(enroll_list);

/*excluding patients younger than 30*/

Data enroll_list; set &enroll_list.;

age = intck('year', birthday, valid_date);

if age < **30** then delete;

rename valid_date = start_date;

RUN;

PROC freq ;

title 'Excluding candidates < 30 years of age';

tables participation;

run;

/*Marking the follow-up period: till the next March 31st*/

DATA data.ID_list_e; SET enroll_list;

format fu_date date9.;

fu_date = intnx('year', start_date, **1**, 'same') -**1**;

if (out_date NE '.') and (fu_date > out_date) then fu_date = out_date;

fu_time = (fu_date - start_date)/**365.25**; /*follow-up time in year*/

if fu_date < start_date then delete;

RUN;

PROC freq ;

title 'Excluding patients dropped out NHI program at the start';

tables participation / norow nopercent;

run;

**%mend** exclusion;

## PSMatching.sas

/*Reference: Coca-Perraillon M. Local and global optimal propensity score matching. InSAS Global Forum 2007 Apr 16 (Vol. 185, pp. 1-9).

http://www2.sas.com/proceedings/forum2007/185-2007.pdf*/

**%macro** PSMatching(datatreatment=, /*treatment dataset*/

datacontrol=, /*control dataset*/

method=, /*method parameter can be NN (nearest available neighbor), caliper or radius.*/

numberofcontrols=, /*number of control matched per treatment*/

caliper=, /*Caliper can be any number indicating the size of the caliper*/

replacement=, /*(yes/no) A control unit could be selected more than once.*/

dataoutput=); /*output dataset*/

/* Create copies of the treated units if N > 1 */;

data _Treatment0(drop= i);

set &datatreatment.;

do i= **1** to &numberofcontrols.;

RandomNumber= ranuni(**12345**);

output;

end;

run;

/* Randomly sort both datasets */

proc sort data= _Treatment0 out= _Treatment(drop= RandomNumber);

by RandomNumber;

run;

data _Control0; set &datacontrol.;

RandomNumber= ranuni(**45678**);

run;

proc sort data= _Control0 out= _Control(drop= RandomNumber);

by RandomNumber;

run;

data Matched(keep = IdSelectedControl MatchedToTreatID);

***length pscoreC 8;

***length idC 8;

/* Load Control dataset into the hash object */

if _N_= **1** then do;

declare hash h(dataset: "_Control", ordered: 'no');

declare hiter iter('h');

h.defineKey('idC');

h.defineData('pscoreC', 'idC');

h.defineDone();

call missing(idC, pscoreC);

end;

/* Open the treatment */

set _Treatment;

%if %upcase(&method) ~= RADIUS %then %do;

retain BestDistance **99**;

%end;

/* Iterate over the hash */

rc= iter.first();

if (rc=**0**) then BestDistance= **99**;

do while (rc = **0**);

/* Caliper */

%if %upcase(&method) = CALIPER %then %do;

if (pscoreT - &caliper) <= pscoreC <= (pscoreT + &caliper) then do;

ScoreDistance = abs(pscoreT - pscoreC);

if ScoreDistance < BestDistance then do;

BestDistance = ScoreDistance;

IdSelectedControl = idC;

MatchedToTreatID = idT;

end;

end;

%end;

/* NN */

%if %upcase(&method) = NN %then %do;

ScoreDistance = abs(pscoreT - pscoreC);

if ScoreDistance < BestDistance then do;

BestDistance = ScoreDistance;

IdSelectedControl = idC;

MatchedToTreatID = idT;

end;

%end;

%if %upcase(&method) = NN or %upcase(&method) = CALIPER %then %do;

rc = iter.next();

/* Output the best control and remove it */

if (rc ~= **0**) and BestDistance ~=**99** then do;

output;

%if %upcase(&replacement) = NO %then %do;

rc1 = h.remove(key: IdSelectedControl);

%end;

end;

%end;

/* Radius */

%if %upcase(&method) = RADIUS %then %do;

if (pscoreT - &caliper) <= pscoreC <= (pscoreT + &caliper) then do;

IdSelectedControl = idC;

MatchedToTreatID = idT;

output;

end;

rc = iter.next();

%end;

end;

run;

/* Delete temporary tables. Quote for debugging */

proc datasets;

delete _:(gennum=all);

run;

/*Construct the matched dataset: &dataoutput.*/

DATA matched; SET matched;

stratum = _N_; /*create stratum as matched ID*/

RUN;

PROC SQL;

CREATE TABLE matched_treated AS

SELECT a.*, b.stratum

FROM &datatreatment. as a, matched as b

WHERE b.MatchedToTreatID = a.idT;

QUIT;

PROC SQL;

CREATE TABLE matched_untreated AS

SELECT a.*, b.stratum

FROM &datacontrol. as a, matched as b

WHERE b.IdSelectedControl = a.idC;

QUIT;

DATA &dataoutput.; SET matched_treated matched_untreated;

drop idT idC pscoreT pscoreC _level_;

run;

**%mend** PSMatching;

## Demographic_analysis 4.1.sas

/*Table 1.demographics: FPICP participants vs. non-participants*/

/* Input parameters:

enroll_list: target file containing the patient list

depend: dependent variable for grouping

Outputs: data.enroll_list_t1 after producing the table 1 (demographics)

*/

**%macro** demographic_analysis(enroll_list, depend);

data enroll_list; set &enroll_list.;

income = ins_amt; if income = **0** then income = '.'; /*ins_amt: monthly income (TWD); if income = 0, then the beneficiary belongs to dependent population*/

run;

/*categorizing age and income; age --> 20-35-50-65-75-85; income --> categorized by tertiles*/

PROC UNIVARIATE DATA = enroll_list noprint;

title 'categorizing income by tertiles';

VAR income;

Output out = summary PCTLPRE=Ter PCTLPTS=**33**,**67**;

RUN;

DATA _null_; set summary;

call symputx('Ter33', Ter33);

call symputx('Ter67', Ter67);

run;

DATA enroll_list; set enroll_list;

/*categorizing income*/

if &Ter33 > income then income_s = "low ";

if &Ter67 > income >= &Ter33 then income_s = "medium";

if income >= &Ter67 then income_s = "high ";

/*categorizing age*/

if **35**>age then age_s = "20~35";

if **50**>age>=**35** then age_s = "35~50";

if **65**>age>=**50** then age_s = "50~65";

if **75**>age>=**65** then age_s = "65~75";

if **85**>age >=**75** then age_s = "75~85";

if age>=**85** then age_s = "85 up";

/*categorizing age: elder or not*/

if **65**>age then elder = "0";

if age>=**65** then elder = "1";

run;

proc freq DATA = enroll_list; /*2*2 table: gender/age/income comparison between groups*/

title 'gender, age, income';

tables sex*&depend. age_s*&depend. income_s*&depend. / norow nopercent chisq; RUN;

proc means n mean std; /*statistics of age comparison between groups*/

title 'age';

class &depend.;

var age;

proc ttest; /*statistics of age comparison between groups, with t-test*/

title 'ttest for age';

class &depend.;

var age;

run;

proc freq DATA = enroll_list; /*2*2 table: Region comparison between groups, DMP4P*/

title 'Regional divisions, DMP4P';

tables branch_id*&depend. DMP4P_group*&depend. / norow nopercent chisq; RUN;

/*acquiring the levels of urbanization (urban) from the area code (area_no_i)*/

/*Sorting the urbanization data containing zip codes and levels of urbanization*/

PROC SORT DATA = source.urban_3_level out = urban_3_level;

BY REG_ZIP_CODE; run;

DATA urban_3_level; SET urban_3_level;

by REG_ZIP_CODE;

ZIP_CODE = put(reg_zip_code,$3.);

if first.REG_ZIP_CODE then output; run;

/*use LEFT JOIN to add "urban" to enroll_list*/

PROC SQL; create table enroll_list as

select a.*, b.urban

from enroll_list as a left join urban_3_level as b /*dataset "urban_3_level" contains the mapping of level of urbanization and the area codes*/

on a.zip_code = b.zip_code; QUIT;

/*go back to enroll_list and check the frequency*/

DATA data.ID_list_e_t1; SET enroll_list;

PROC FREQ; /*2*2 table: level of urbanization*/

title 'level of urbanization';

tables urban * &depend. / norow nopercent chisq;

RUN;

/*statistics of the follow-up time and age; summing up the follow-up person-year*/

PROC MEANS N MIN MEAN STD MAX Q1 MEDIAN Q3 SUM data = data.ID_list_e_t1;

title 'statistics of the follow-up time and age; summing up the follow-up person-year';

var fu_time age; run;

**%mend** demographic_analysis;

## Before_regression_simple 4.1.sas

/* Parameters:

ID_list: target file containing the ID list

output_file: the name of the resulting output file for subsequent regression

*/

**%macro** before_regression_simple(ID_list, output_file);

DATA &output_file.; set &ID_list.;

time = (fu_date - start_date)/**365.25**; /*The unit of time-to-event is "year" in the regression*/

if fu_time < **0** then delete;

if age >= **65** then elder = **1**; else elder = **0**; run; /*defining "elder" as age >= 65*/

run;

**%mend** before_regression_simple;

## Assess_oo_target1 DM 4.1.sas

**%MACRO** assess_oo_target1();

/*source.DM_exam_wide: NHI codes for diabetes examinations

A1C: 09006C

Fundus (FE): 23501C, 23502C

LDL: 09044C

MAU: 12111C, 27065B

Urine (UR): 06012C, 06013C

*/

DATA OO_target; SET source.DM_exam_wide;

run;

/*creating macro: Create a 'look up' list of NHI code(s) for each diabetes examination*/

**%macro** ootarget(var);

%global &var;

proc sql;

select quote(trim(&var.)) into :&var

separated by " "

from OO_target

where &var. is not missing;

quit;

**%mend** ootarget;

/*For diagnoses in ootarget: Create 'look up' lists*/

ods select none; /*close ODS function to prevent printing*/

%***ootarget***(A1C); /*09006C */

%***ootarget***(Fundus); /*23501C, 23502C */

%***ootarget***(MAU); /*12111C, 27065B */

%***ootarget***(LDL); /*09044C */

%***ootarget***(Urine); /*06012C, 06013C */

ods select all; /*reopen ODS function*/

/*Identifying target in OO files

Input variables:

OO: D103 files (OO) are datasets containing details of ambulatory care orders; the suffix denotes the date (YYYYMM)

num: the sequential numbering of the file (one OO file for every month starting from 2010; therefore, the numberings of OO files in year 2015 and 2016 are 60~84)

Outputs:

OO_[DM exam]_[numbering]: Filtering each D103 file by each diabetes examination

*/

**%macro** ooidentify(OO, num);

DATA

OO_A1C_&num.

OO_Fundus_&num.

OO_MAU_&num.

OO_LDL_&num.

OO_Urine_&num.

;

SET &oo.;

tid = FEE_YM || hosp_id || APPL_TYPE || APPL_DATE || CASE_TYPE || SEQ_NO;

/* the components of “tid” (index for matching datasets)

FEE_YM: the date of OPD visit (YYYY-MM)

hosp_id: the ID of hospital or clinic

APPL_TYPE: the type of reimbursement claim

APPL_DATE: the date of reimbursement application

CASE_TYPE: the type of OPD visit

SEQ_NO: sequence number

*/

if order_code in :(&A1C) then output OO_A1C_&num.;

if order_code in :(&Fundus) then output OO_Fundus_&num.;

if order_code in :(&MAU) then output OO_MAU_&num.;

if order_code in :(&LDL) then output OO_LDL_&num.;

if order_code in :(&Urine) then output OO_Urine_&num.;

KEEP order_code tid FEE_YM hosp_id APPL_TYPE APPL_DATE CASE_TYPE SEQ_NO;

RUN;

**%mend** ooidentify;

%***ooidentify***(m.d103_201501, **61**);

%***ooidentify***(m.d103_201502, **62**);

%***ooidentify***(m.d103_201503, **63**);

%***ooidentify***(m.d103_201504, **64**);

%***ooidentify***(m.d103_201505, **65**);

%***ooidentify***(m.d103_201506, **66**);

%***ooidentify***(m.d103_201507, **67**);

%***ooidentify***(m.d103_201508, **68**);

%***ooidentify***(m.d103_201509, **69**);

%***ooidentify***(m.d103_201510, **70**);

%***ooidentify***(m.d103_201511, **71**);

%***ooidentify***(m.d103_201512, **72**);

%***ooidentify***(m.d103_201601, **73**);

%***ooidentify***(m.d103_201602, **74**);

%***ooidentify***(m.d103_201603, **75**);

%***ooidentify***(m.d103_201604, **76**);

%***ooidentify***(m.d103_201605, **77**);

%***ooidentify***(m.d103_201606, **78**);

%***ooidentify***(m.d103_201607, **79**);

%***ooidentify***(m.d103_201608, **80**);

%***ooidentify***(m.d103_201609, **81**);

%***ooidentify***(m.d103_201610, **82**);

%***ooidentify***(m.d103_201611, **83**);

%***ooidentify***(m.d103_201612, **84**);

**%mend** assess_oo_target1;

## Assess_oo_target2 DM 4.1.sas

/*input parameters:

Din: dataset with IDs of target population

Dout: the output dataset

name: specify the name of diabetes examination

Output: ID list of target population, along with frequency of diabetes examinations

*/

**%macro** assess_oo_target2(Din, Dout, name);

DATA id_list; SET &Din.; run;

/*from OO to CD*/

**%macro** OO_to_CD(OO, cd_num, CD); /*For outputs from last procedure (OO_[DM exam]_[numbering]: filtering each D103 file by each diabetes examination), match them to the D102 files (Ambulatory care expenditures by visits) to extract the patient’s ID*/

DATA tmp_CD; SET &CD.;

tid = FEE_YM || hosp_id || APPL_TYPE || APPL_DATE || CASE_TYPE || SEQ_NO;

KEEP id func_date tid FEE_YM hosp_id APPL_TYPE APPL_DATE CASE_TYPE SEQ_NO;

RUN;

%local serial; /*&serial is for numbering of the temporary OO files.*/

%do i= **1** %to **6**;

%let serial = %eval((&cd_num-1)*6+&i); /* The serial can be derived from cd_num, which is the numbering of cd-oo sets. E.g. (cd_num = 1) refers to d102_2010_hy1 and temporary OO files numbered 1~6*/

PROC SQL; CREATE TABLE CD&OO.&serial. AS

SELECT a.order_code, a.tid, b.id, b.func_date, b.tid

FROM &OO.&serial. as a inner join tmp_CD as b

ON a.tid = b.tid;

QUIT;

%end;

**%mend** OO_to_CD;

%***OO_to_CD***(OO_&name._,**11**, m.d102_2015_hy1); /*OO_&name._61~66 inner join d102_2015_hy1*/

%***OO_to_CD***(OO_&name._,**12**, m.d102_2015_hy2); /*OO_&name._67~72 inner join d102_2015_hy2*/

%***OO_to_CD***(OO_&name._,**13**, m.d102_2016_hy1); /*OO_&name._73~78 inner join d102_2016_hy1*/

%***OO_to_CD***(OO_&name._,**14**, m.d102_2016_hy2); /*OO_&name._79~84 inner join d102_2016_hy2*/

/*Combining the CDOO_&name._# to form CDOOtarget: dataset containing all relevant OPD visits with patient ID and the diabetes examinations received*/

**DATA** CDOOtarget; SET CDOO_&name._61-CDOO_&name._84;

**run**;

/*calculate the frequency of diabetes examinations for each ID*/

**proc** **means** n data=CDOOtarget noprint;

class id;

output out=CDOO_N n=n;

**run**;

/*matching the frequency of diabetes examinations for each ID back to the target population dataset*/

**PROC** **SQL**; CREATE table &Dout. AS

SELECT a.*, b.n as &name.

FROM id_list as a left join CDOO_N as b

on a.id = b.id;

**QUIT**;

**%mend** assess_oo_target2;

## Num_of_event_table 4.1.sas

/*Reporting numbers of events or summary measures over time.*/

/*Input parameters

ID_list: target file containing the ID list to calculate the of events

Output: numbers of events and followed person-years for the event

*/

**%macro** num_of_event_table(ID_list);

DATA ID_list; SET &ID_list.;

FPICP_DMP4P = cat(participation, DMP4P_group);

array DM_exam A1c--Urine;

do over DM_exam;

if DM_exam = '.' then DM_exam = **0**;

DM_exam = DM_exam/time;

end;

Proc means mean LCLM UCLM data=ID_list;

title 'grouped means of DM exams';

class elder ;

var A1c--Urine A1c_group--Urine_group; run;

Proc means mean data=ID_list;

class sex ;

var A1c--Urine A1c_group--Urine_group; run;

Proc means mean data=ID_list;

class CCI ;

var A1c--Urine A1c_group--Urine_group; run;

Proc means mean data=ID_list;

class FPICP_DMP4P ;

var A1c--Urine A1c_group--Urine_group; run;

Proc anova data=ID_list;

title 'ANOVA of DM exams';

class elder sex CCI FPICP_DMP4P;

model A1c--Urine A1c_group--Urine_group = elder sex CCI FPICP_DMP4P;

means FPICP_DMP4P / BON ; /*ANOVA with post hoc bonferroni test*/

run;

**%mend** num_of_event_table;

## Logistic_data 4.1.sas

/*Logistic regression

dependent variable: outcomes (denoted as "depend")

explanatory variables: FPICP member

confounders: age, gender, comorbidities*/

/* Input parameter:

ID_list: target file containing the time, the outcome, and the variables.

depend: the dependent variable

*/

**%macro** Logistic_data(ID_list, depend);

/*Logistic regression: crude OR*/

proc logistic data = &ID_list.;

title 'Logistic regression: crude OR';

strata stratum;

class participation (ref = '0') / param = ref;

class DMP4P_group (ref = '0') / param = ref;

model &depend.(event='1') = participation DMP4P_group;

run;

/*Logistic regression: adjusted OR*/

proc logistic data = &ID_list.;

title 'Logistic regression: adjusted OR';

strata stratum;

class elder (ref = '0') / param = ref; /*confounders: age, gender, CCI*/

class sex (ref = '2') / param = ref;

class CCI (ref = 'low ') / param = ref;

class participation (ref = '0') / param = ref;

class DMP4P_group (ref = '0') / param = ref;

model &depend.(event='1') = elder sex CCI participation DMP4P_group;

run;

/*Logistic regression: adjusted OR with interaction*/

proc logistic data = &ID_list.;

title 'Logistic regression: adjusted OR';

strata stratum;

class elder (ref = '0') / param = ref; /*confounders: age, gender, CCI*/

class sex (ref = '2') / param = ref;

class CCI (ref = 'low ') / param = ref;

class participation (ref = '0') / param = ref;

class DMP4P_group (ref = '0') / param = ref;

model &depend.(event='1') = elder sex CCI participation DMP4P_group participation*DMP4P_group;

run;

**%mend** Logistic_data;

# Appendix 5. Level of urbanization according to the region code in Taiwan

Filename: urban_3_level.sas7bdat

Description: This dataset comprises region codes of the residential region and corresponding levels of urbanization according to previous publication, with level 1 referring to the “most urbanized” and level 3 referring to the “least urbanized” communities.

Coding of variables:

- Dist_name: Name of the residential region (in Chinese)
- REG_ZIP_CODE: Postal code
- Area_no: Region code
- Urban: Level of urbanization

Reference:

Liu, C.-Y., et al., Incorporating development stratification of Taiwan townships into sampling design of large scale health interview survey. J Health Manag, 2006. 4(1): p. 1-22.

| *Dist_Name* | *REG_ZIP_CODE* | *Urban* | *area_no* |
| --- | --- | --- | --- |
| *台北市* | *.* | *high* | *100* |
| *台北市松山區* | *105* | *high* | *101* |
| *台北市大安區* | *106* | *high* | *102* |
| *台北市大同區* | *103* | *high* | *109* |
| *台北市中山區* | *104* | *high* | *110* |
| *台北市內湖區* | *114* | *medium* | *111* |
| *台北市南港區* | *115* | *medium* | *112* |
| *台北市士林區* | *111* | *medium* | *115* |
| *台北市北投區* | *112* | *medium* | *116* |
| *台北市信義區* | *110* | *high* | *117* |
| *台北市中正區* | *100* | *high* | *118* |
| *台北市萬華區* | *108* | *high* | *119* |
| *台北市文山區* | *116* | *medium* | *120* |
| *高雄市* | *.* | *high* | *200* |
| *高雄市鹽埕區* | *803* | *high* | *201* |
| *高雄市鼓山區* | *804* | *medium* | *202* |
| *高雄市左營區* | *813* | *medium* | *203* |
| *高雄市楠梓區* | *811* | *medium* | *204* |
| *高雄市三民區* | *807* | *low* | *205* |
| *高雄市新興區* | *800* | *high* | *206* |
| *高雄市前金區* | *801* | *high* | *207* |
| *高雄市苓雅區* | *802* | *high* | *208* |
| *高雄市前鎮區* | *806* | *medium* | *209* |
| *高雄市旗津區* | *805* | *high* | *210* |
| *高雄市小港區* | *812* | *medium* | *211* |
| *基隆市* | *.* | *medium* | *1100* |
| *基隆市中正區* | *202* | *medium* | *1101* |
| *基隆市七堵區* | *206* | *medium* | *1102* |
| *基隆市暖暖區* | *205* | *medium* | *1103* |
| *基隆市仁愛區* | *200* | *medium* | *1104* |
| *基隆市中山區* | *203* | *medium* | *1105* |
| *基隆市安樂區* | *204* | *medium* | *1106* |
| *基隆市信義區* | *201* | *medium* | *1107* |
| *新竹市* | *.* | *medium* | *1200* |
| *新竹市東區* | *300* | *medium* | *1201* |
| *新竹市北區* | *300* | *medium* | *1204* |
| *新竹市香山區* | *300* | *medium* | *1205* |
| *台中市* | *.* | *high* | *1700* |
| *台中市中區* | *400* | *high* | *1701* |
| *台中市東區* | *401* | *medium* | *1702* |
| *台中市西區* | *403* | *high* | *1703* |
| *台中市南區* | *402* | *high* | *1704* |
| *台中市北區* | *404* | *high* | *1705* |
| *台中市西屯區* | *407* | *medium* | *1706* |
| *台中市南屯區* | *408* | *medium* | *1707* |
| *台中市北屯區* | *406* | *medium* | *1708* |
| *台南市* | *.* | *medium* | *2100* |
| *台南市東區* | *701* | *high* | *2101* |
| *台南市南區* | *702* | *medium* | *2102* |
| *台南市西區* | *.* | *medium* | *2103* |
| *台南市北區* | *704* | *medium* | *2104* |
| *台南市中區* | *.* | *high* | *2105* |
| *台南市安南區* | *709* | *medium* | *2106* |
| *台南市安平區* | *708* | *medium* | *2107* |
| *台南市中西區* | *534* | *high* | *2108* |
| *嘉義市* | *.* | *medium* | *2200* |
| *嘉義市東區* | *600* | *medium* | *2201* |
| *嘉義市西區* | *600* | *medium* | *2202* |
| *台北縣* | *.* | *low* | *3100* |
| *台北縣板橋市* | *220* | *high* | *3101* |
| *台北縣三重市* | *241* | *high* | *3102* |
| *台北縣永和市* | *234* | *high* | *3103* |
| *台北縣中和市* | *235* | *high* | *3104* |
| *台北縣新店市* | *231* | *medium* | *3105* |
| *台北縣新莊市* | *242* | *high* | *3106* |
| *台北縣樹林鎮* | *238* | *medium* | *3107* |
| *台北縣鶯歌鎮* | *239* | *medium* | *3108* |
| *台北縣三峽鎮* | *237* | *low* | *3109* |
| *台北縣淡水鎮* | *251* | *medium* | *3110* |
| *台北縣汐止鎮* | *221* | *medium* | *3111* |
| *台北縣瑞芳鎮* | *224* | *low* | *3112* |
| *台北縣土城鄉* | *236* | *low* | *3113* |
| *台北縣蘆洲鄉* | *247* | *low* | *3114* |
| *台北縣五股鄉* | *248* | *medium* | *3115* |
| *台北縣泰山鄉* | *243* | *medium* | *3116* |
| *台北縣林口鄉* | *244* | *medium* | *3117* |
| *台北縣深坑鄉* | *222* | *medium* | *3118* |
| *台北縣石碇鄉* | *223* | *low* | *3119* |
| *台北縣坪林鄉* | *232* | *low* | *3120* |
| *台北縣三芝鄉* | *252* | *low* | *3121* |
| *台北縣石門鄉* | *253* | *low* | *3122* |
| *台北縣八里鄉* | *249* | *medium* | *3123* |
| *台北縣平溪鄉* | *226* | *low* | *3124* |
| *台北縣雙溪鄉* | *227* | *low* | *3125* |
| *台北縣貢寮鄉* | *228* | *low* | *3126* |
| *台北縣金山鄉* | *208* | *low* | *3127* |
| *台北縣萬里鄉* | *207* | *low* | *3128* |
| *台北縣烏來鄉* | *233* | *low* | *3129* |
| *桃園縣* | *.* | *medium* | *3200* |
| *桃園縣桃園市* | *330* | *medium* | *3201* |
| *桃園縣中壢市* | *320* | *medium* | *3202* |
| *桃園縣大溪鎮* | *335* | *low* | *3203* |
| *桃園縣楊梅鎮* | *326* | *medium* | *3204* |
| *桃園縣蘆竹鄉* | *338* | *medium* | *3205* |
| *桃園縣大園鄉* | *337* | *medium* | *3206* |
| *桃園縣龜山鄉* | *333* | *high* | *3207* |
| *桃園縣八德鄉* | *334* | *low* | *3208* |
| *桃園縣龍潭鄉* | *325* | *medium* | *3209* |
| *桃園縣平鎮鄉* | *324* | *low* | *3210* |
| *桃園縣新屋鄉* | *327* | *low* | *3211* |
| *桃園縣觀音鄉* | *328* | *low* | *3212* |
| *桃園縣復興鄉* | *336* | *low* | *3213* |
| *新竹縣* | *.* | *low* | *3300* |
| *新竹縣關西鎮* | *306* | *low* | *3301* |
| *新竹縣新埔鎮* | *305* | *low* | *3302* |
| *新竹縣竹東鎮* | *310* | *medium* | *3303* |
| *新竹縣竹北市* | *302* | *medium* | *3305* |
| *新竹縣湖口鄉* | *303* | *medium* | *3306* |
| *新竹縣橫山鄉* | *312* | *low* | *3307* |
| *新竹縣新豐鄉* | *304* | *medium* | *3308* |
| *新竹縣芎林鄉* | *307* | *low* | *3309* |
| *新竹縣寶山鄉* | *308* | *low* | *3310* |
| *新竹縣北埔鄉* | *314* | *low* | *3311* |
| *新竹縣峨眉鄉* | *315* | *low* | *3312* |
| *新竹縣尖石鄉* | *313* | *low* | *3313* |
| *新竹縣五峰鄉* | *311* | *low* | *3314* |
| *宜蘭縣* | *.* | *low* | *3400* |
| *宜蘭縣宜蘭市* | *260* | *medium* | *3401* |
| *宜蘭縣羅東鎮* | *265* | *medium* | *3402* |
| *宜蘭縣蘇澳鎮* | *270* | *low* | *3403* |
| *宜蘭縣頭城鎮* | *261* | *low* | *3404* |
| *宜蘭縣礁溪鄉* | *262* | *low* | *3405* |
| *宜蘭縣壯圍鄉* | *263* | *low* | *3406* |
| *宜蘭縣員山鄉* | *264* | *low* | *3407* |
| *宜蘭縣冬山鄉* | *269* | *low* | *3408* |
| *宜蘭縣五結鄉* | *268* | *low* | *3409* |
| *宜蘭縣三星鄉* | *266* | *low* | *3410* |
| *宜蘭縣大同鄉* | *267* | *low* | *3411* |
| *宜蘭縣南澳鄉* | *272* | *low* | *3412* |
| *苗栗縣* | *.* | *low* | *3500* |
| *苗栗縣苗栗市* | *360* | *medium* | *3501* |
| *苗栗縣苑裡鎮* | *358* | *low* | *3502* |
| *苗栗縣通霄鎮* | *357* | *low* | *3503* |
| *苗栗縣竹南鎮* | *350* | *medium* | *3504* |
| *苗栗縣頭份鎮* | *351* | *low* | *3505* |
| *苗栗縣後龍鎮* | *356* | *low* | *3506* |
| *苗栗縣卓蘭鎮* | *369* | *low* | *3507* |
| *苗栗縣大湖鄉* | *364* | *low* | *3508* |
| *苗栗縣公館鄉* | *363* | *low* | *3509* |
| *苗栗縣銅鑼鄉* | *366* | *low* | *3510* |
| *苗栗縣南庄鄉* | *353* | *low* | *3511* |
| *苗栗縣頭屋鄉* | *362* | *low* | *3512* |
| *苗栗縣三義鄉* | *367* | *low* | *3513* |
| *苗栗縣西湖鄉* | *368* | *low* | *3514* |
| *苗栗縣造橋鄉* | *361* | *low* | *3515* |
| *苗栗縣三灣鄉* | *352* | *low* | *3516* |
| *苗栗縣獅潭鄉* | *354* | *low* | *3517* |
| *苗栗縣泰安鄉* | *365* | *low* | *3518* |
| *台中縣* | *.* | *low* | *3600* |
| *台中縣豐原市* | *420* | *low* | *3601* |
| *台中縣東勢鎮* | *423* | *low* | *3602* |
| *台中縣大甲鎮* | *437* | *low* | *3603* |
| *台中縣清水鎮* | *436* | *medium* | *3604* |
| *台中縣沙鹿鎮* | *433* | *medium* | *3605* |
| *台中縣梧棲鎮* | *435* | *medium* | *3606* |
| *台中縣后里鄉* | *421* | *low* | *3607* |
| *台中縣神岡鄉* | *429* | *medium* | *3608* |
| *台中縣潭子鄉* | *427* | *medium* | *3609* |
| *台中縣大雅鄉* | *428* | *medium* | *3610* |
| *台中縣新社鄉* | *426* | *low* | *3611* |
| *台中縣石岡鄉* | *422* | *low* | *3612* |
| *台中縣外埔鄉* | *438* | *low* | *3613* |
| *台中縣大安鄉* | *439* | *low* | *3614* |
| *台中縣烏日鄉* | *414* | *medium* | *3615* |
| *台中縣大肚鄉* | *432* | *medium* | *3616* |
| *台中縣龍井鄉* | *434* | *medium* | *3617* |
| *台中縣霧峰鄉* | *413* | *medium* | *3618* |
| *台中縣太平鄉* | *411* | *low* | *3619* |
| *台中縣大里鄉* | *412* | *low* | *3620* |
| *台中縣和平鄉* | *424* | *low* | *3621* |
| *彰化縣* | *.* | *low* | *3700* |
| *彰化縣彰化市* | *500* | *medium* | *3701* |
| *彰化縣鹿港鎮* | *505* | *medium* | *3702* |
| *彰化縣和美鎮* | *508* | *medium* | *3703* |
| *彰化縣北斗鎮* | *521* | *low* | *3704* |
| *彰化縣員林鎮* | *510* | *low* | *3705* |
| *彰化縣溪湖鎮* | *514* | *low* | *3706* |
| *彰化縣田中鎮* | *520* | *low* | *3707* |
| *彰化縣二林鎮* | *526* | *low* | *3708* |
| *彰化縣線西鄉* | *507* | *low* | *3709* |
| *彰化縣伸港鄉* | *509* | *medium* | *3710* |
| *彰化縣福興鄉* | *506* | *low* | *3711* |
| *彰化縣秀水鄉* | *504* | *low* | *3712* |
| *彰化縣花壇鄉* | *503* | *medium* | *3713* |
| *彰化縣芬園鄉* | *502* | *low* | *3714* |
| *彰化縣大村鄉* | *515* | *medium* | *3715* |
| *彰化縣埔鹽鄉* | *516* | *low* | *3716* |
| *彰化縣埔心鄉* | *513* | *low* | *3717* |
| *彰化縣永靖鄉* | *512* | *low* | *3718* |
| *彰化縣社頭鄉* | *511* | *low* | *3719* |
| *彰化縣二水鄉* | *530* | *low* | *3720* |
| *彰化縣田尾鄉* | *522* | *low* | *3721* |
| *彰化縣埤頭鄉* | *523* | *low* | *3722* |
| *彰化縣芳苑鄉* | *528* | *low* | *3723* |
| *彰化縣大城鄉* | *527* | *low* | *3724* |
| *彰化縣竹塘鄉* | *525* | *low* | *3725* |
| *彰化縣溪州鄉* | *524* | *low* | *3726* |
| *南投縣* | *.* | *low* | *3800* |
| *南投縣南投市* | *540* | *low* | *3801* |
| *南投縣埔里鎮* | *545* | *low* | *3802* |
| *南投縣草屯鎮* | *542* | *low* | *3803* |
| *南投縣竹山鎮* | *557* | *low* | *3804* |
| *南投縣集集鎮* | *552* | *low* | *3805* |
| *南投縣名間鄉* | *551* | *low* | *3806* |
| *南投縣鹿谷鄉* | *558* | *low* | *3807* |
| *南投縣中寮鄉* | *541* | *low* | *3808* |
| *南投縣魚池鄉* | *555* | *low* | *3809* |
| *南投縣國姓鄉* | *544* | *low* | *3810* |
| *南投縣水里鄉* | *553* | *low* | *3811* |
| *南投縣信義鄉* | *556* | *low* | *3812* |
| *南投縣仁愛鄉* | *546* | *low* | *3813* |
| *雲林縣* | *.* | *low* | *3900* |
| *雲林縣斗六市* | *640* | *medium* | *3901* |
| *雲林縣斗南鎮* | *630* | *low* | *3902* |
| *雲林縣虎尾鎮* | *632* | *low* | *3903* |
| *雲林縣西螺鎮* | *648* | *low* | *3904* |
| *雲林縣土庫鎮* | *633* | *low* | *3905* |
| *雲林縣北港鎮* | *651* | *low* | *3906* |
| *雲林縣古坑鄉* | *646* | *low* | *3907* |
| *雲林縣大埤鄉* | *631* | *low* | *3908* |
| *雲林縣莿桐鄉* | *647* | *low* | *3909* |
| *雲林縣林內鄉* | *643* | *low* | *3910* |
| *雲林縣二崙鄉* | *649* | *low* | *3911* |
| *雲林縣崙背鄉* | *637* | *low* | *3912* |
| *雲林縣麥寮鄉* | *638* | *low* | *3913* |
| *雲林縣東勢鄉* | *635* | *low* | *3914* |
| *雲林縣褒忠鄉* | *634* | *low* | *3915* |
| *雲林縣台西鄉* | *636* | *low* | *3916* |
| *雲林縣元長鄉* | *655* | *low* | *3917* |
| *雲林縣四湖鄉* | *654* | *low* | *3918* |
| *雲林縣口湖鄉* | *653* | *low* | *3919* |
| *雲林縣水林鄉* | *652* | *low* | *3920* |
| *嘉義縣* | *.* | *low* | *4000* |
| *嘉義縣朴子鎮* | *613* | *low* | *4001* |
| *嘉義縣布袋鎮* | *625* | *low* | *4002* |
| *嘉義縣大林鎮* | *622* | *low* | *4003* |
| *嘉義縣民雄鄉* | *621* | *medium* | *4004* |
| *嘉義縣溪口鄉* | *623* | *low* | *4005* |
| *嘉義縣新港鄉* | *616* | *low* | *4006* |
| *嘉義縣六腳鄉* | *615* | *low* | *4007* |
| *嘉義縣東石鄉* | *614* | *low* | *4008* |
| *嘉義縣義竹鄉* | *624* | *low* | *4009* |
| *嘉義縣鹿草鄉* | *611* | *low* | *4010* |
| *嘉義縣太保鄉* | *612* | *low* | *4011* |
| *嘉義縣水上鄉* | *608* | *low* | *4012* |
| *嘉義縣中埔鄉* | *606* | *low* | *4013* |
| *嘉義縣竹崎鄉* | *604* | *low* | *4014* |
| *嘉義縣梅山鄉* | *603* | *low* | *4015* |
| *嘉義縣番路鄉* | *602* | *low* | *4016* |
| *嘉義縣大埔鄉* | *607* | *low* | *4017* |
| *嘉義縣阿里山* | *605* | *low* | *4018* |
| *台南縣* | *.* | *low* | *4100* |
| *台南縣新營市* | *730* | *medium* | *4101* |
| *台南縣鹽水鎮* | *737* | *low* | *4102* |
| *台南縣白河鎮* | *732* | *low* | *4103* |
| *台南縣麻豆鎮* | *721* | *low* | *4104* |
| *台南縣佳里鎮* | *722* | *low* | *4105* |
| *台南縣新化鎮* | *712* | *low* | *4106* |
| *台南縣善化鎮* | *741* | *low* | *4107* |
| *台南縣學甲鎮* | *726* | *low* | *4108* |
| *台南縣柳營鄉* | *736* | *low* | *4109* |
| *台南縣後壁鄉* | *731* | *low* | *4110* |
| *台南縣東山鄉* | *733* | *low* | *4111* |
| *台南縣下營鄉* | *735* | *low* | *4112* |
| *台南縣六甲鄉* | *734* | *low* | *4113* |
| *台南縣官田鄉* | *720* | *low* | *4114* |
| *台南縣大內鄉* | *742* | *low* | *4115* |
| *台南縣西港鄉* | *723* | *low* | *4116* |
| *台南縣七股鄉* | *724* | *low* | *4117* |
| *台南縣將軍鄉* | *725* | *low* | *4118* |
| *台南縣北門鄉* | *727* | *low* | *4119* |
| *台南縣新市鄉* | *744* | *low* | *4120* |
| *台南縣安定鄉* | *745* | *low* | *4121* |
| *台南縣山上鄉* | *743* | *low* | *4122* |
| *台南縣玉井鄉* | *714* | *low* | *4123* |
| *台南縣楠西鄉* | *715* | *low* | *4124* |
| *台南縣南化鄉* | *716* | *low* | *4125* |
| *台南縣左鎮鄉* | *713* | *low* | *4126* |
| *台南縣仁德鄉* | *717* | *low* | *4127* |
| *台南縣歸仁鄉* | *711* | *medium* | *4128* |
| *台南縣關廟鄉* | *718* | *low* | *4129* |
| *台南縣龍崎鄉* | *719* | *low* | *4130* |
| *台南縣永康鄉* | *710* | *low* | *4131* |
| *高雄縣* | *.* | *medium* | *4200* |
| *高雄縣鳳山市* | *830* | *medium* | *4201* |
| *高雄縣岡山鎮* | *820* | *medium* | *4202* |
| *高雄縣旗山鎮* | *842* | *low* | *4203* |
| *高雄縣美濃鎮* | *843* | *low* | *4204* |
| *高雄縣林園鄉* | *832* | *medium* | *4205* |
| *高雄縣大寮鄉* | *831* | *medium* | *4206* |
| *高雄縣大樹鄉* | *840* | *medium* | *4207* |
| *高雄縣仁武鄉* | *814* | *medium* | *4208* |
| *高雄縣大社鄉* | *815* | *medium* | *4209* |
| *高雄縣鳥松鄉* | *833* | *high* | *4210* |
| *高雄縣橋頭鄉* | *825* | *medium* | *4211* |
| *高雄縣燕巢鄉* | *824* | *low* | *4212* |
| *高雄縣田寮鄉* | *823* | *low* | *4213* |
| *高雄縣阿蓮鄉* | *822* | *low* | *4214* |
| *高雄縣路竹鄉* | *821* | *medium* | *4215* |
| *高雄縣湖內鄉* | *829* | *medium* | *4216* |
| *高雄縣茄萣鄉* | *852* | *medium* | *4217* |
| *高雄縣永安鄉* | *828* | *low* | *4218* |
| *高雄縣彌陀鄉* | *827* | *medium* | *4219* |
| *高雄縣梓官鄉* | *826* | *medium* | *4220* |
| *高雄縣六龜鄉* | *844* | *low* | *4221* |
| *高雄縣甲仙鄉* | *847* | *low* | *4222* |
| *高雄縣杉林鄉* | *846* | *low* | *4223* |
| *高雄縣內門鄉* | *845* | *low* | *4224* |
| *高雄縣茂林鄉* | *851* | *low* | *4225* |
| *高雄縣桃源鄉* | *848* | *low* | *4226* |
| *高雄縣那瑪夏* | *849* | *low* | *4227* |
| *屏東縣* | *.* | *low* | *4300* |
| *屏東縣屏東市* | *900* | *medium* | *4301* |
| *屏東縣潮州鎮* | *920* | *low* | *4302* |
| *屏東縣東港鎮* | *928* | *low* | *4303* |
| *屏東縣恆春鎮* | *946* | *low* | *4304* |
| *屏東縣萬丹鄉* | *913* | *low* | *4305* |
| *屏東縣長治鄉* | *908* | *low* | *4306* |
| *屏東縣麟洛鄉* | *909* | *low* | *4307* |
| *屏東縣九如鄉* | *904* | *low* | *4308* |
| *屏東縣里港鄉* | *905* | *low* | *4309* |
| *屏東縣鹽埔鄉* | *907* | *low* | *4310* |
| *屏東縣高樹鄉* | *906* | *low* | *4311* |
| *屏東縣萬巒鄉* | *923* | *low* | *4312* |
| *屏東縣內埔鄉* | *912* | *low* | *4313* |
| *屏東縣竹田鄉* | *911* | *low* | *4314* |
| *屏東縣新埤鄉* | *925* | *low* | *4315* |
| *屏東縣枋寮鄉* | *940* | *low* | *4316* |
| *屏東縣新園鄉* | *932* | *low* | *4317* |
| *屏東縣崁頂鄉* | *924* | *low* | *4318* |
| *屏東縣林邊鄉* | *927* | *low* | *4319* |
| *屏東縣南州鄉* | *926* | *low* | *4320* |
| *屏東縣佳冬鄉* | *931* | *low* | *4321* |
| *屏東縣琉球鄉* | *929* | *low* | *4322* |
| *屏東縣車城鄉* | *944* | *low* | *4323* |
| *屏東縣滿州鄉* | *947* | *low* | *4324* |
| *屏東縣枋山鄉* | *941* | *low* | *4325* |
| *屏東縣三地門* | *901* | *low* | *4326* |
| *屏東縣霧台鄉* | *902* | *low* | *4327* |
| *屏東縣瑪家鄉* | *903* | *low* | *4328* |
| *屏東縣泰武鄉* | *921* | *low* | *4329* |
| *屏東縣來義鄉* | *922* | *low* | *4330* |
| *屏東縣春日鄉* | *942* | *low* | *4331* |
| *屏東縣獅子鄉* | *943* | *low* | *4332* |
| *屏東縣牡丹鄉* | *945* | *low* | *4333* |
| *澎湖縣* | *.* | *low* | *4400* |
| *澎湖縣馬公市* | *880* | *low* | *4401* |
| *澎湖縣湖西鎮* | *885* | *low* | *4402* |
| *澎湖縣白沙鄉* | *884* | *low* | *4403* |
| *澎湖縣西嶼鄉* | *881* | *low* | *4404* |
| *澎湖縣望安鄉* | *882* | *low* | *4405* |
| *澎湖縣七美鄉* | *883* | *low* | *4406* |
| *花蓮縣* | *.* | *low* | *4500* |
| *花蓮縣花蓮市* | *970* | *medium* | *4501* |
| *花蓮縣鳳林鎮* | *975* | *low* | *4502* |
| *花蓮縣玉里鎮* | *981* | *low* | *4503* |
| *花蓮縣新城鄉* | *971* | *medium* | *4504* |
| *花蓮縣吉安鄉* | *973* | *medium* | *4505* |
| *花蓮縣壽豐鄉* | *974* | *low* | *4506* |
| *花蓮縣光復鄉* | *976* | *low* | *4507* |
| *花蓮縣豐濱鄉* | *977* | *low* | *4508* |
| *花蓮縣瑞穗鄉* | *978* | *low* | *4509* |
| *花蓮縣富里鄉* | *983* | *low* | *4510* |
| *花蓮縣秀林鄉* | *972* | *low* | *4511* |
| *花蓮縣萬榮鄉* | *979* | *low* | *4512* |
| *花蓮縣卓溪鄉* | *982* | *low* | *4513* |
| *台東縣* | *.* | *low* | *4600* |
| *台東縣台東市* | *950* | *low* | *4601* |
| *台東縣成功鎮* | *961* | *low* | *4602* |
| *台東縣關山鎮* | *956* | *low* | *4603* |
